# Investigating the Prevalence, Gender Predilection, and Inheritance Patterns of Genodermatoses: A Tertiary Hospital Study

**DOI:** 10.7759/cureus.62455

**Published:** 2024-06-15

**Authors:** Namratha Puttur, Asmita Kapoor, Kshitiz Lakhey, Aravind Reddy, Nishtha Malik, Shubham Deokar

**Affiliations:** 1 Dermatology, Venereology and Leprosy, Dr. D. Y. Patil Medical College, Hospital & Research Centre, Dr. D. Y. Patil Vidyapeeth (Deemed to be University), Pune, IND

**Keywords:** tertiary hospital-based study, epidemiology, genes, genetics, dermatology

## Abstract

Genodermatoses encompass a spectrum of hereditary skin disorders stemming from mutations in genes pivotal for skin development, structure, and function. This study investigated the prevalence, gender predilection, and inheritance patterns of genodermatoses in a tertiary-level hospital through a one-year observational study. Among 157,051 dermatology outpatient department patients, 105 cases of genodermatoses were diagnosed, yielding a prevalence rate of 0.067%. Hamartoneoplastic syndromes and inherited disorders of cornification were the most prevalent subgroups, with neurofibromatosis type 1 and tuberous sclerosis complex 1 leading within these categories. The average age at presentation varied among different subgroups. A 2:1 male-to-female ratio was observed across all subgroups. Autosomal dominant inheritance was predominant. A positive family history in 46 cases and consanguinity among parents in 28 instances was reported. Genodermatoses pose diagnostic challenges due to their clinical complexity and rarity, which is compounded by limited epidemiological data. Molecular diagnosis advancements offer insights into genotype-phenotype correlations and facilitate genetic counseling and prenatal diagnosis (PND). Raising awareness among healthcare professionals and the public is critical for improving the quality of life for affected individuals.

## Introduction

Genodermatoses are hereditary conditions characterized by genetically mediated abnormalities affecting the skin and its appendages. These disorders arise from mutations in genes crucial for skin development, structure, and function, resulting in a diverse array of clinical manifestations encompassing skin color, texture, hair, nails, sweat glands, mucous membranes, and internal organs. Commonly affected internal organs include the kidneys, heart, and lungs. For instance, tuberous sclerosis can lead to kidney cysts and angiomyolipomas, while Sturge-Weber syndrome may cause cerebral atrophy and glaucoma. Some conditions manifest at birth, while others emerge later in life. They may range in severity from minor cosmetic issues to profound, debilitating ailments [1,2]. Hence, genodermatoses frequently necessitate specialized medical management and care from dermatologists, geneticists, and other healthcare specialists [3]. However, the paucity of data available on the prevalence of genodermatoses in the Indian subcontinent may lead to medical professionals missing the diagnosis of these rare conditions [4].

Understanding the epidemiology of genodermatoses is critical for designing effective management strategies and providing genetic counselling. This clinical epidemiological study aimed to investigate the prevalence, gender predilection, and inheritance patterns of genodermatoses in a tertiary-level hospital.

## Materials and methods

A hospital-based observational study was conducted at Dr. D. Y. Patil Medical College, Hospital, and Research Centre, Pune, India, over a one-year period from March 2023 to March 2024. The study included patients of all ages attending the dermatology outpatient department (OPD) who were diagnosed with genodermatoses based on clinical manifestations. Ten patients who did not consent to be part of the study were excluded from it.

A detailed history was taken after obtaining informed consent/assent from all participants and recording their socio-demographic information. This included information on skin complaints, their onset, progression, family history, exacerbating and relieving factors, and any associated medical disorders. Pedigree analysis was performed for all patients based on the collected history.

A comprehensive clinical examination was conducted to identify any cutaneous or extracutaneous abnormalities. Routine blood examinations were performed, including complete blood count with differential count, erythrocyte sedimentation rate, liver function tests, renal function tests, and urine routine and microscopic examination. Radiological examinations were conducted as necessary.

Data on patient demographics, mode of inheritance, and family history were collected and analyzed. Statistical analysis was performed using Statistical Product and Service Solutions (SPSS, version 20.0; SPSS Inc., Chicago, IL) software. Continuous variables were presented as mean ± standard deviation (SD), and categorical variables were presented as absolute numbers. A p-value of less than 0.05 was considered statistically significant.

## Results

Among the 157,051 patients attending the dermatology outpatient department, 115 patients were diagnosed with genodermatoses. Twenty-five patients had been previously diagnosed at other centers, although records were unavailable for verification. Thirty patients had symptoms since childhood but did not seek medical attention. The delay in treatment was primarily because of a lack of access to specialized care, insufficient awareness, and socioeconomic barriers. Ten patients were excluded from the study because of a lack of consent. Therefore, 105 patients were included in our study, resulting in a prevalence rate of 0.067% and an incidence rate of 0.051%. All 105 patients underwent routine blood investigations. Sixty-two patients underwent skin biopsies for diagnosis. Fifty-six patients underwent radiological examinations in the form of X-rays and MRIs. Thirty-four out of 105 patients underwent genetic testing, but the number was limited because of financial constraints. Forty-three patients were diagnosed solely based on clinical examination.

**Figure 1:**
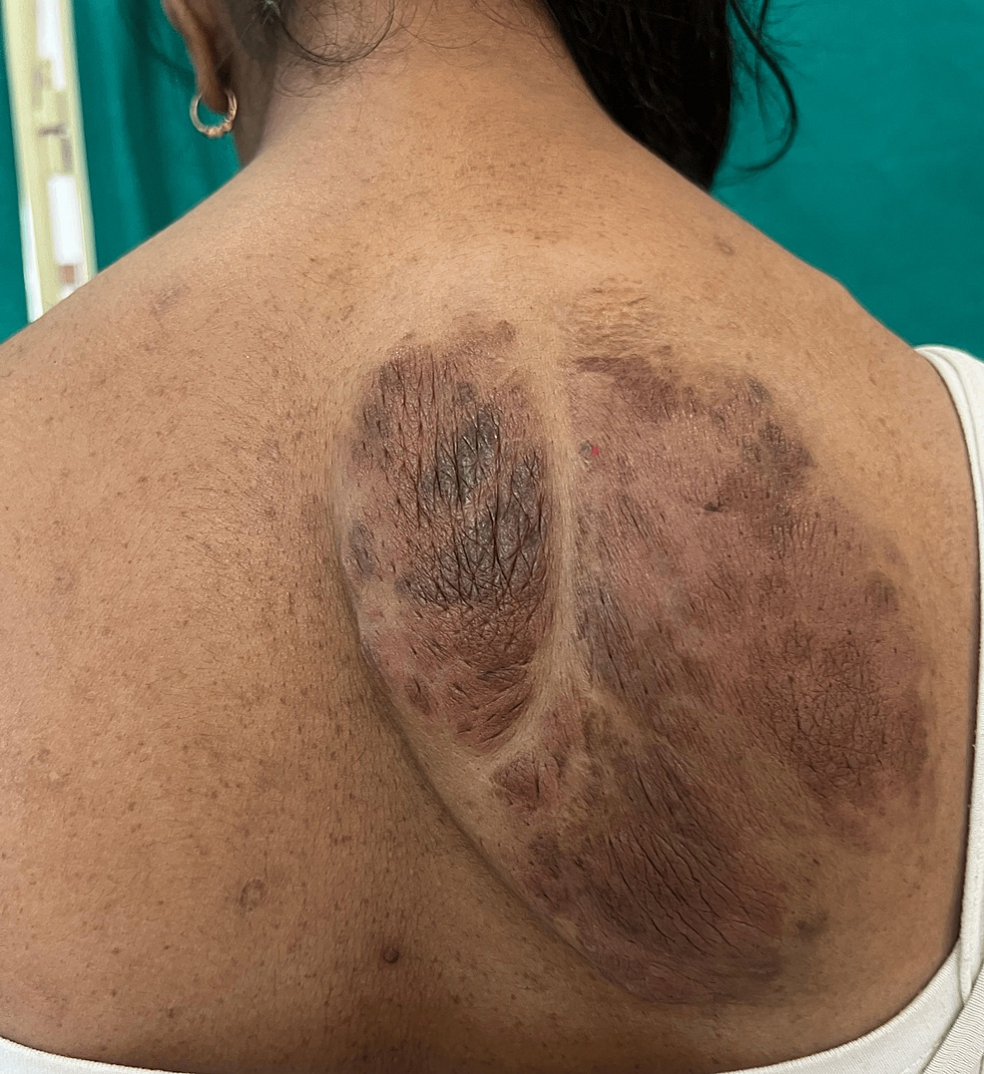
Plexiform neurofibroma in a case of neurofibromatosis type 1. A 21-year-old female patient who fulfilled the National Institutes of Health criteria for neurofibromatosis type 1 with eight cafe-au-lait macules, a single plexiform neurofibroma, 10 neurofibromas, as well as axillary and inguinal freckling.

**Figure 2:**
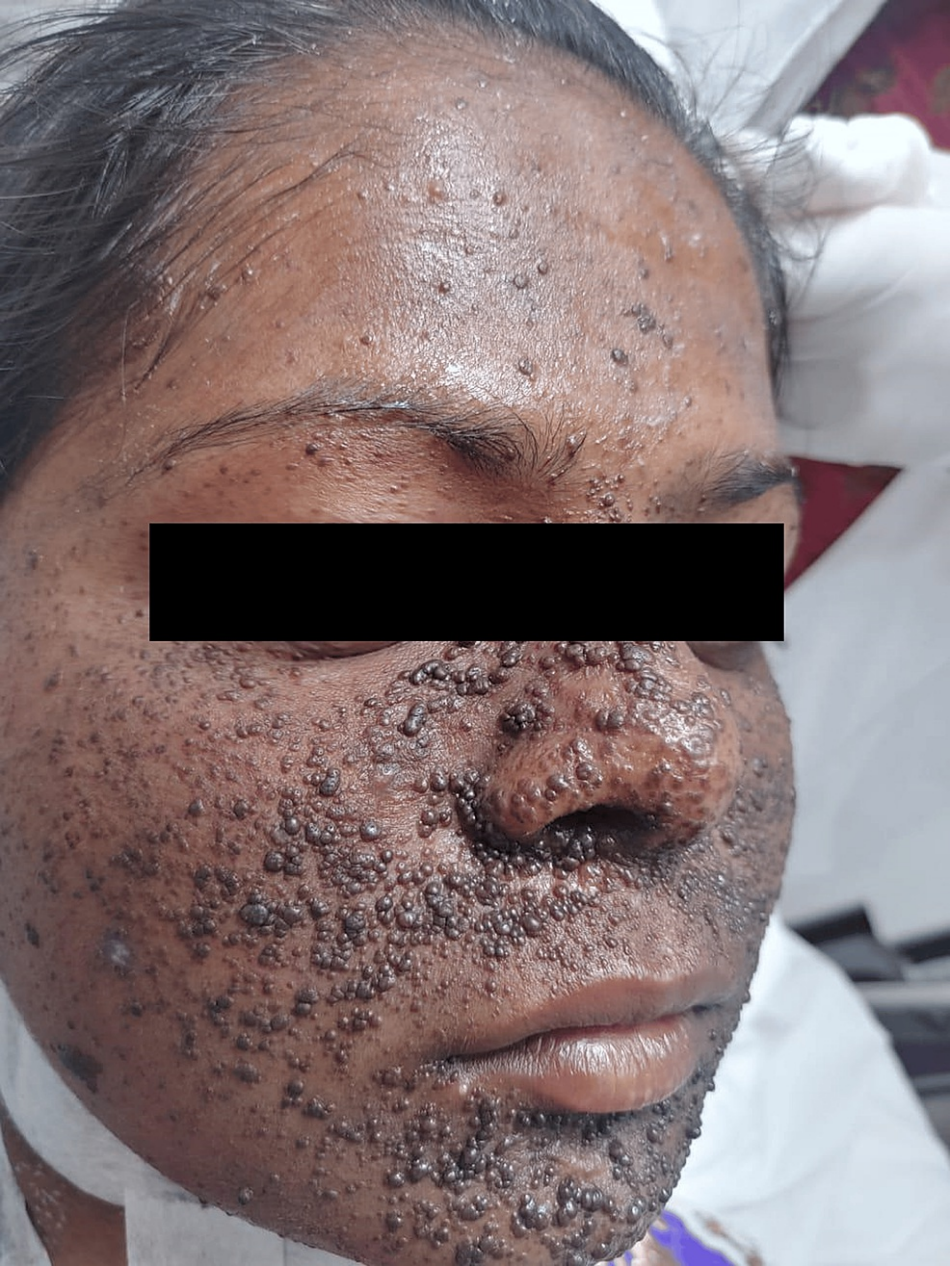
Adenoma sebaceum over the face. A 16-year-old female patient presented with clinical features like adenoma sebaceum (facial angiofibromas) and a shagreen patch. She was diagnosed as a case of tuberous sclerosis complex 1.

**Figure 3:**
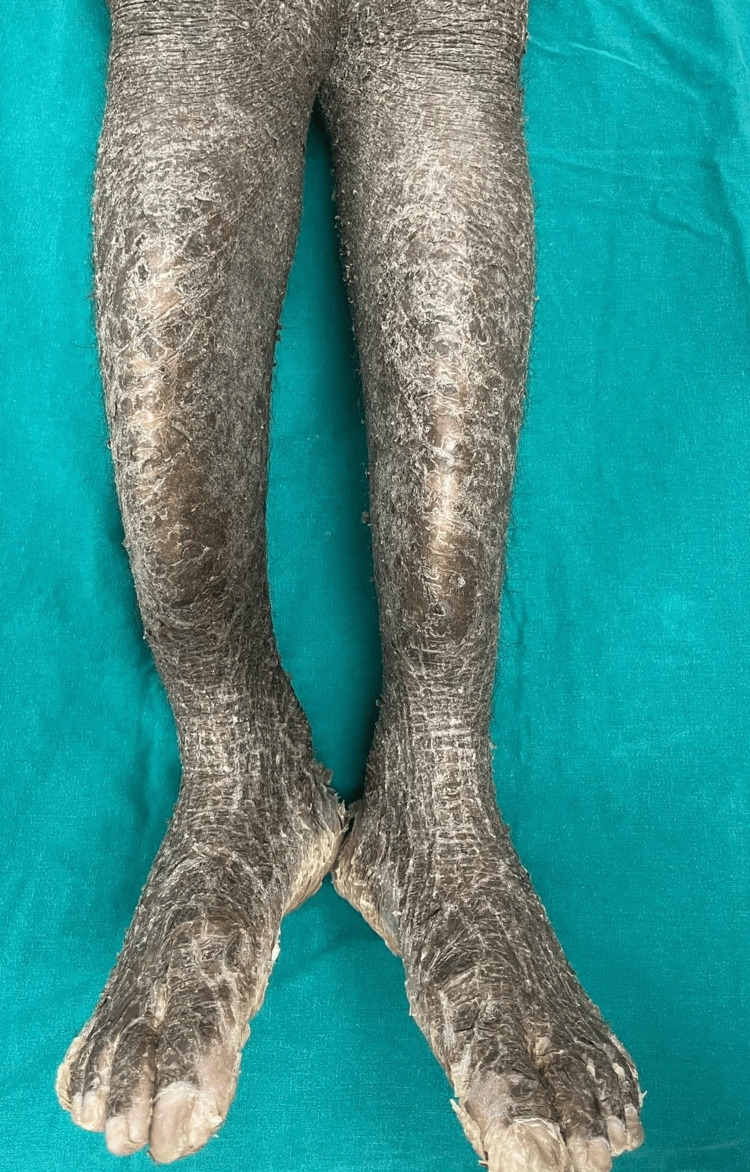
Plate-like scales in a case of lamellar ichthyosis. A 17-year-old patient presented with severe palmoplantar keratoderma and plate-like scales all over his body. His parents recalled that he was born encased in a thin shiny membrane. A history of consanguinity among the parents was present. No previous documents of diagnosis were available, and the patient was newly diagnosed as a case of lamellar ichthyosis at our center.

The most prevalent subgroup was hamartoneoplastic syndromes, which comprised 32 cases. This was followed by inherited disorders of certification, with 25 cases being recorded (Table 1). Neurofibromatosis type 1 (Figure 1) and tuberous sclerosis complex 1 (Figure 2) were the most common syndromes within these subgroups as they accounted for 18 and 12 cases, respectively. Additionally, neurofibromatosis type 2 was identified in two cases. The age at presentation varied across different genodermatoses subgroups. Overall, the average age of presentation across all cases was 23.55 ± 2.28 years. Inherited disorders of cornification, such as ichthyosis vulgaris and lamellar ichthyosis (Figure 3), typically presented at a younger age compared to hamartoneoplastic syndromes. The distinct clinical features and varying prevalence of inherited acantholytic and blistering disorders, genetic disorders of pigmentation, and ectodermal dysplasia were also represented. Inherited metabolic disorders, such as mitochondrial syndrome, presented at a later average age of 32 years compared to other subgroups. The observed male-to-female ratio was 2:1, which indicated a notable gender predilection across all genodermatoses subgroups.

**Table 1:** Table of demographics for 105 patients with genodermatoses. Summary of demographics for 105 patients with genodermatoses at Dr. D. Y. Patil Medical College over one year. It includes the number of individuals (N), average age at presentation (mean ± SD), and gender distribution (male/female). Statistical analysis was performed using SPSS version 20.0. Continuous variables are represented as mean ± SD, while categorical variables are represented as numbers. Significance is set at p < 0.05. MHTFR: methylenetetrahydrofolate reductase

Genodermatoses	N (no. of individuals)	Male	Female	Age at presentation to the outpatient department
Hamartoneoplastic syndromes	32	17	15	29.75±3.839
· Neurofibromatosis type 1	18	10	8	34.16±5.06
· Tuberous sclerosis complex 1	12	7	5	24.91±4.82
· Neurofibromatosis type 2	2	0	2	19.00±5.54
Inherited disorders of cornification	25	21	5	25.14±4.73
· Ichthyosis vulgaris	9	6	3	21.44±5.01
· Lamellar ichthyosis	5	5	0	21.80±5.35
· Congenital ichthyosiform erythroderma	4	3	1	14.75±7.14
· X-linked recessive ichthyosis	2	2	0	22.00±1.38
· Autosomal recessive congenital ichthyosis	1	1	0	21.00
· Bullous congenital ichthyosiform erythroderma	1	1	0	7.00
· Epidermolytic ichthyosis	1	1	0	7.00
· Epidermolytic palmoplantar keratoderma	1	0	1	10.00
· Erythrokeratoderma variabilis	1	1	0	6.00
Inherited acantholytic and blistering disorders	9	2	7	24.92±6.27
· Darier disease	5	2	3	21.40±1.96
· Hailey disease	2	0	2	25.00±5.54
· Acrokeratosis verruciformis of Hopf	1		1	5.00
· Epidermolysis bullosa simplex	1		1	10.00
Genetic disorders of pigmentation	8	6	2	22.92±5.48
· Oculo cutaneous albinism	3	2	1	18.67±1.41
· Dowling degos	2	2		32.00±2.77
· Dyschromatosis universalis hereditaria	1	0	1	19.00
· Hermansky pudlack	1	1	0	7.00
· Incontinentia pigmenti	1	1	0	10.00
Ectodermal dysplasia	7	3	4	26.85±7.37
· Hypohydrotic ectodermal dysplasia	7	3	4	26.85±7.37
Inherited metabolic disorders	5	5	0	32.20±16.44
· Mitochondrial syndrome	2	2	0	47.00±20.78
· Hyperhomocysteinemia with heterozygous MHTFR mutation	1	1	0	35.00
· Menke disease	1	1	0	29.00
· Methylmalonic acidemia	1	1	0	3.00
Inherited immunodeficiencies	4	2	1	20.25±6.76
· Severe combined immunodeficiency syndrome	3	2	1	20.66±8.97
· Chediak Higashi syndrome	1	1		19.00
Tumours of skin appendages	4	2	2	33.75±17.32
· Brooke Spiegler syndrome	2	0	2	19.50±20.09
· Multiple familial trichoepithelioma (type 1)	2	2	0	48.00±4.15
DNA repair disorders with cutaneous features	3	1	2	24.00±3.69
· Xeroderma pigmentosum	3	1	2	24.00±3.69
Inherited hair disorders	3	1	2	13.33±2.82
· Woolly hair syndrome	3	1	2	13.33±2.82
Genetic disorders of collagen, elastin, dermal matrix	2	2	0	20.00±2.77
· Alport syndrome	2	2	0	20.00±2.77
Others	1	1	0	25.00
· Steatocystoma multiplex	1	1	0	25.00
Syndromes with premature aging	1	1	0	8.00
· Progeroid syndrome	1	1	0	8.00
Grand total	105	64	41	23.55±2.28

Autosomal dominant inheritance was the most common mode, with 54 cases, followed by autosomal recessive inheritance with 27 cases. Only one case of X-linked dominant inheritance was observed (Table 2). A positive family history was recorded in 46 patients, with consanguinity among parents reported in 28 cases.

**Table 2:** Mode of inheritance of genodermatoses. Summary of the mode of inheritance for 105 patients with genodermatoses at Dr. D. Y. Patil Medical College. The table lists the number of individuals (N) for each inheritance pattern: autosomal dominant (54), autosomal recessive (27), semi-dominant (9), mitochondrial (2), X-linked recessive (12), and X-linked dominant (1). Statistical analysis was performed using SPSS version 20.0.

Mode of inheritance	Number of individuals
Autosomal dominant	54
Autosomal recessive	27
Semi-dominant	9
Mitochondrial	2
X-linked recessive	12
X-linked dominant	1

## Discussion

Genodermatoses present significant challenges for patients and healthcare providers due to their rarity and complex clinical presentations. These conditions arise from mutations in genes crucial for skin development, structure, and function [1,2]. Specialized medical management and care are required to address the wide array of clinical symptoms caused by these mutations [3].

The complexity inherent in the diverse spectrum of genodermatoses often leads to delays and uncertainties in diagnosis. However, significant strides in molecular diagnosis have deepened our understanding of genodermatoses [5,6]. Identifying genetic mutations and determining gene function are crucial for prognosis and informed management [7]. This enables appropriate genetic counseling and deoxyribonucleic acid (DNA)-based PND for high-risk families [8].

PND for inherited skin disorders is essential for cases involving previously affected pregnancies, familial history, carrier status, or abnormal prenatal screening. The techniques currently available include fetal skin biopsy, amniocentesis, chorionic villus sampling (CVS), ultrasonography (USG), maternal serum screening, and cell-free fetal DNA and messenger ribonucleic acid (mRNA) analysis. Each of these modalities has distinct timings, risks, and advantages. A fetal skin biopsy, performed at 16-20 weeks, is invaluable when the causative gene or mutation is unknown. However, it carries risks such as fetal scarring and a 1%-3% chance of fetal loss. Amniocentesis, performed at 15-20 weeks, is widely practiced but may cause amniotic fluid leakage and infection. It is also associated with a 0.5% risk of fetal loss. CVS at 10-12 weeks allows earlier diagnosis but carries a 1% risk of fetal death and potential limb defects. USG is non-invasive and useful throughout pregnancy but may only reveal disease features late. It also lacks well-established diagnostic criteria for many conditions. Maternal serum screening at 15-20 weeks is non-invasive but requires confirmatory testing, especially for X-linked ichthyosis. Cell-free fetal DNA and mRNA, which constitute about 3%-6% of genetic material in maternal plasma, are ideally analyzed between four and 10 weeks. However, isolating fetal DNA is challenging. This limits its use to mutations inherited from the father [9-11].

Despite advances in DNA-based PND, pregnancy termination remains common for affected fetuses. The decision to pursue PND hinges on religious beliefs, ethics, personal values, and disease severity. Ethical dilemmas arise regarding the severity of the disease justifying PND and subsequent termination of pregnancy. Conditions such as harlequin ichthyosis and some epidermolysis bullosa variants pose life-threatening risks while others such as neurofibromatosis and tuberous sclerosis cause severe disfigurement and systemic issues. However, many genodermatoses, which are mild, raise ethical debates about termination. Pre-implantation genetic diagnosis offers an alternative by testing embryos post-in vitro fertilization (IVF) and implanting only the unaffected ones [10].

Recent advances in the management of genodermatoses have not been limited to screening and diagnosis. There have been strides in tailoring treatments according to specific genetic mutations and molecular pathways of each disease. For example, medications targeting various signaling pathways, such as mitogen-activated protein kinase (MEK), B-rapidly accelerated fibrosarcoma (B-RAF), mammalian target of rapamycin (mTOR), transforming growth factor-beta (TGF-β), and Janus kinase/signal transducers and activators of transcription (JAK-STAT) and rat sarcoma (RAS) pathways, are being evaluated in cases of neurofibromatosis type 1 (NF1). Similarly, drugs that affect mTOR signaling pathways have shown promise in tuberous sclerosis complex 1 (TSC1). Ustekinumab is being used to target the skewed interleukin-23 (IL-23)/T-helper cell 17 (Th17) axis in ichthyosis to alleviate inflammation. For severe cases such as harlequin ichthyosis, inhibitors of nitric oxide synthase 2 (NOS2) or JAK are being explored to restore lipid barriers. In hypohidrotic ectodermal dysplasia, protein replacement therapies such as Fc-ectodysplasin A (Fc-EDA) fusion protein help enhance gland and tooth development in prenatal care. Furthermore, gene therapies utilizing tools such as clustered regularly interspaced short palindromic repeats (CRISPR)-associated protein 9 (CRISPR/Cas9) offer a precise approach to correcting genetic anomalies seen in conditions such as keratinopathies. Base editing techniques provide a nuanced method to rectify single-base mutations [12].

By definition, rare diseases are conditions that occur in fewer than one in 2,000 people in any WHO region [13]. While most genodermatoses fall under the category of rare diseases, their exact incidence or prevalence is frequently unknown. The observed prevalence rate of 0.067% and the incidence rate of 0.051% highlight the relative rarity of these conditions within the studied population. The later average age of presentation seen in inherited metabolic disorders, such as mitochondrial syndrome, suggests distinct clinical trajectories for different genodermatoses. The male-to-female ratio of 2:1 seen in our study also highlights a notable gender disparity across all subgroups and reflects potential genetic and hormonal influences on disease expression. The study also observed a notable number of cases where consanguinity among parents was reported. In clinical genetics, consanguineous marriage refers to a union between two individuals who are related as second cousins or closer, resulting in an inbreeding coefficient (F) equal to or greater than 0.0156. Consanguineous marriages can increase the likelihood of an offspring inheriting deleterious genetic variants responsible for genodermatoses [14].

Our research has helped identify the common genodermatoses within our population. Increased awareness would help dermatologists and clinicians from various other specialties such as obstetrics, pediatrics, and internal medicine ensure early diagnosis and appropriate management of these diseases. This timely intervention has the potential to improve the quality of life of patients while drastically reducing their financial and emotional burdens.

Despite these insights, our study has certain limitations. Its retrospective nature and reliance on clinical diagnoses without molecular confirmation may introduce bias. Additionally, its generalizability may be limited as it is a single-center study with a relatively small sample size. The timeframe of only one year further restricts the scope of findings. Further research with larger sample sizes and extended study periods is essential to validate findings and deepen our understanding of the epidemiology of genodermatoses.

## Conclusions

The rarity of genodermatoses and the lack of awareness surrounding them pose significant challenges in diagnosing patients in this specialized field. Our study provides detailed data on the demographics of various genodermatoses inherent in the population. This information equips clinicians to develop better diagnostic strategies and research superior treatment modalities.

## References

[1] Pandhi D (2015). Current status of genodermatoses: an Indian perspective. Indian J Dermatol Venereol Leprol.

[2] Lim X, Nusse R (2013). Wnt signaling in skin development, homeostasis, and disease. Cold Spring Harb Perspect Biol.

[3] Parker JC, Rangu S, Grand KL, Bhoj EJ, Castelo-Soccio L, Sheppard SE (2021). Genetic skin disorders: the value of a multidisciplinary clinic. Am J Med Genet A.

[4] Dalave K, Deora MS, Sabhandasani S, Singh P, Mittal A, Shah B (2019). Clinico-epidemiological study of genodermatoses in pediatric age group. Int J Res Dermatol.

[5] Schaffer JV (2012). Molecular diagnostics in genodermatoses. Semin Cutan Med Surg.

[6] Hiremagalore RN, Nizamabad N, Kamasamudram V (2008). Molecular diagnostics in genodermatoses - simplified. Indian J Dermatol Venereol Leprol.

[7] Chiu FP, Doolan BJ, McGrath JA, Onoufriadis A (2021). A decade of next-generation sequencing in genodermatoses: the impact on gene discovery and clinical diagnostics. Br J Dermatol.

[8] Williams ML (1986). Ichthyosis: genetic heterogeneity, genodermatoses, and genetic counseling. Arch Dermatol.

[9] Gautam M, Ali F (2021). Prenatal diagnosis in dermatology. Indian J Paediatr Dermatol.

[10] Luu M, Cantatore-Francis JL, Glick SA (2010). Prenatal diagnosis of genodermatoses: current scope and future capabilities. Int J Dermatol.

[11] Ashton GHS, Eady RAJ, McGrath JA (2000). Prenatal diagnosis for inherited skin diseases. Clin Dermatol.

[12] Morren MA, Legius E, Giuliano F, Hadj-Rabia S, Hohl D, Bodemer C (2021). Challenges in treating genodermatoses: new therapies at the horizon. Front Pharmacol.

[13] The Lancet Global Health (2024). The landscape for rare diseases in 2024. Lancet Global Health.

[14] Bittles A (2001). Consanguinity and its relevance to clinical genetics. Clin Genet.

